# Feature tracking compared with tissue tagging measurements of segmental strain by cardiovascular magnetic resonance

**DOI:** 10.1186/1532-429X-16-10

**Published:** 2014-01-22

**Authors:** LiNa Wu, Tjeerd Germans, Ahmet Güçlü, Martijn W Heymans, Cornelis P Allaart, Albert C van Rossum

**Affiliations:** 1 Department of Cardiology, VU University Medical Center, Amsterdam, The Netherlands; 2 Institute for Cardiovascular Research, Amsterdam, The Netherlands; 3 Interuniversity Cardiology Institute of the Netherlands (ICIN), Utrecht, The Netherlands; 4 Department of Epidemiology and Biostatistics, VU University Medical Center, Amsterdam, The Netherlands

**Keywords:** Cardiovascular magnetic resonance, Myocardial wall motion, Tissue tagging, Myocardial feature-tracking

## Abstract

**Background:**

Left ventricular segmental wall motion analysis is important for clinical decision making in cardiac diseases. Strain analysis with myocardial tissue tagging is the non-invasive gold standard for quantitative assessment, however, it is time-consuming. Cardiovascular magnetic resonance myocardial feature-tracking (CMR-FT) can rapidly perform strain analysis, because it can be employed with standard CMR cine-imaging. The aim is to validate segmental peak systolic circumferential strain (peak SCS) and time to peak systolic circumferential strain (T2P-SCS) analysed by CMR-FT against tissue tagging, and determine its intra and inter-observer variability.

**Methods:**

Patients in whom both cine CMR and tissue tagging has been performed were selected. CMR-FT analysis was done using endocardial (CMR-FT_endo_) and mid-wall contours (CMR-FT_mid_). The Intra Class Correlation Coefficient (ICC) and Pearson correlation were calculated.

**Results:**

10 healthy volunteers, 10 left bundle branch block (LBBB) and 10 hypertrophic cardiomyopathy patients were selected. With CMR-FT all 480 segments were analyzable and with tissue tagging 464 segments.

Significant differences in mean peak SCS values of the total study group were present between CMR-FT_endo_ and tissue tagging (-23.8 ± 9.9% vs -13.4 ± 3.3%, p < 0.001). Differences were smaller between CMR-FT_mid_ and tissue tagging (-16.4 ± 6.1% vs -13.4 ± 3.3%, p = 0.001). The ICC of the mean peak SCS of the total study group between CMR-FT_endo_ and tissue tagging was low (0.19 (95%-CI-0.10-0.49), p = 0.02). Comparable results were seen between CMR-FT_mid_ and tissue tagging. In LBBB patients, mean T2P-SCS values measured with CMR-FT_endo_ and CMR-FT_mid_ were 418 ± 66 ms, 454 ± 60 ms, which were longer than with tissue tagging, 376 ± 55 ms, both p < 0.05. ICC of the mean T2P-SCS between CMR-FT_endo_ and tissue tagging was 0.64 (95%-CI-0.36-0.81), p < 0.001, this was better in the healthy volunteers and LBBB group, whereas the ICC between CMR-FT_mid_ and tissue tagging was lower.

The intra and inter-observer agreement of segmental peak SCS with CMR-FT_mid_ was lower compared with tissue tagging; similar results were seen for segmental T2P-SCS.

**Conclusions:**

The intra and inter-observer agreement of segmental peak SCS and T2P-SCS is substantially lower with CMR-FT_mid_ compared with tissue tagging. Therefore, current segmental CMR-FT_mid_ techniques are not yet applicable for clinical and research purposes.

## Background

Left ventricular (LV) wall motion analysis is one of the key arbitrators in clinical decision making in ischemic heart disease and cardiomyopathy [1]. Various imaging modalities can be employed for this purpose, such as Doppler echocardiography [2], scintigraphy [3] and cardiovascular magnetic resonance (CMR) [4]. With CMR, wall motion analysis can be performed with steady-state free precession (SSFP) cine-imaging. However, strain analysis has shown to be superior to wall motion analysis to detect differences in myocardial deformation and to determine timing of contraction. Segmental strain analysis can be performed with echocardiography using speckle tracking and with CMR using myocardial tissue tagging with harmonic phase (HARP) imaging.

Myocardial tissue tagging is a sophisticated technique to quantitatively analyse regional intramyocardial deformation and has an excellent inter and intra-observer agreement [4-7]. Although generally appreciated for its incremental value in clinical decision making, CMR segmental strain analysis has not yet become clinical standard because of its elaborate acquisition and post processing [4,8]. Therefore, an alternative, less time-consuming method is desirable. Recently, CMR myocardial feature-tracking (CMR-FT) on standard SSFP cine-images has been developed in order to meet the need for a fast, quantitative assessment of the myocardial segmental strain analysis [9,10]. Since CMR-FT is based on CMR SSFP cine images, no additional sequences are required and the post processing time is importantly reduced while LV contours only have to be drawn in the mid-wall of the myocardium in the end-diastolic phase of the SA cine images.

CMR-FT has recently been validated for global strain analysis [11] and for segmental strain analysis in healthy volunteers [12]. However, data on the accuracy of CMR-FT in patients expected to have segmental abnormalities in both peak strain and timing of deformation is sparse. Therefore, the aim of the present study is to validate segmental circumferential strain and time to peak circumferential strain analysed by CMR-FT with tissue tagging, and to determine its intra and inter-observer reliability in various patient groups.

## Methods

### Patient population

This was a single center, retrospective study. Patients in whom both CMR cine-imaging and tissue tagging had been performed, were selected from our local CMR database. Three study groups were selected. One group of patients with complete left bundle branch block (LBBB) and heart failure was selected; a second group of patients with hypertrophic obstructive cardiomyopathy (HCM) amendable for septal alcohol ablation or myectomy and a third group existed of healthy volunteers, who had no cardiovascular history, no risk factors nor used medication. Patients were excluded when > 50% of the tissue tagging data was un-analysable.

### Cardiovascular magnetic resonance acquisition

CMR studies were performed on a 1.5-Tesla whole body scanner (Magnetom Sonata, Siemens, Erlangen, Germany), using a six-channel phased-array body coil.

SSFP cines were acquired in a single breath hold during mild expiration for 8–10 seconds.

After survey scans, a retrospective triggered balanced SSFP gradient-echo sequence was used for cine-imaging. Typical image parameters were: slice thickness 5 mm, slice gap 5 mm, temporal resolution < 50 ms, repetition time 3.2 ms, echo time 1.54 ms, flip angle 60 degrees and a typical image resolution of 1.3 by 1.6 mm. The number of phases within the cardiac cycle was set at 20.

Myocardial tissue tagging was performed with an ECG gated, multiple breath hold, balanced SSFP line tagging sequence with linear start-up angle for complementary spatial modulation of magnetization (CSPAMM) [13]. Image parameters were: 7 mm slice thickness, temporal resolution of 14.1 ms, repetition time 4.7 ms, echo time of 2.3 ms, flip angle 20 degrees, and image resolution of 1.2 by 3.8 mm, with a tag spacing of 7 mm.

Short-axis (SA) tissue tagging was performed on 3 levels of the LV, positioned at 25%, 50% and 75% of the distance between the mitral valve annulus and the apex on a LV 4-chamber view in end-systole. Acquisition time per slice was approximately 3–4 minutes.

### Cardiovascular magnetic resonance feature-tracking

**Figure 1:**
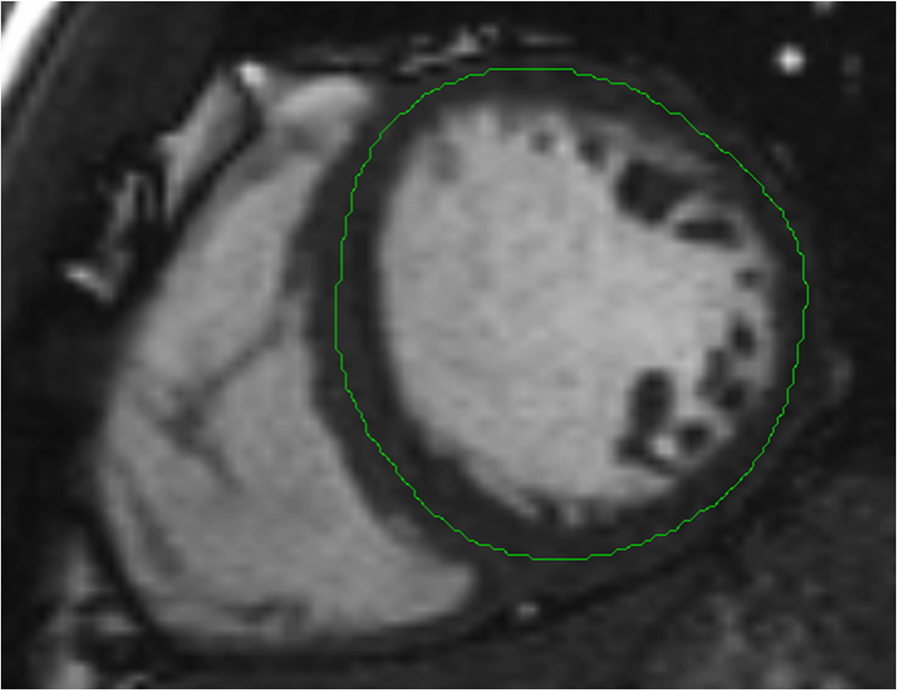
A short-axis image with a contour drawn in the mid-wall of the left ventricle.

CMR-FT was done by Diogenes CMR-FT software (TomTec Imaging Systems, Munich, Germany). LV contours were drawn on the endocardial wall of the myocardium (CMR-FT_endo_) on basal, mid and apical level, as described previously [9]. Since, most circumferential fibers are located in the mid-wall of the LV [14]. CMR-FT was also performed on the same slice position at mid-wall level (CMR-FT_mid_) (Figure 1). The CMR-FT software propagates the contour automatically and follows the motion of the contour throughout the whole cardiac cycle [9]. The contours were checked and when necessary manually adjusted. Peak SCS and T2P-SCS values of both CMR-FT_endo_ and CMR-FT_mid_ were compared with tissue tagging.

### Post-processing tissue tagging

**Figure 2:**
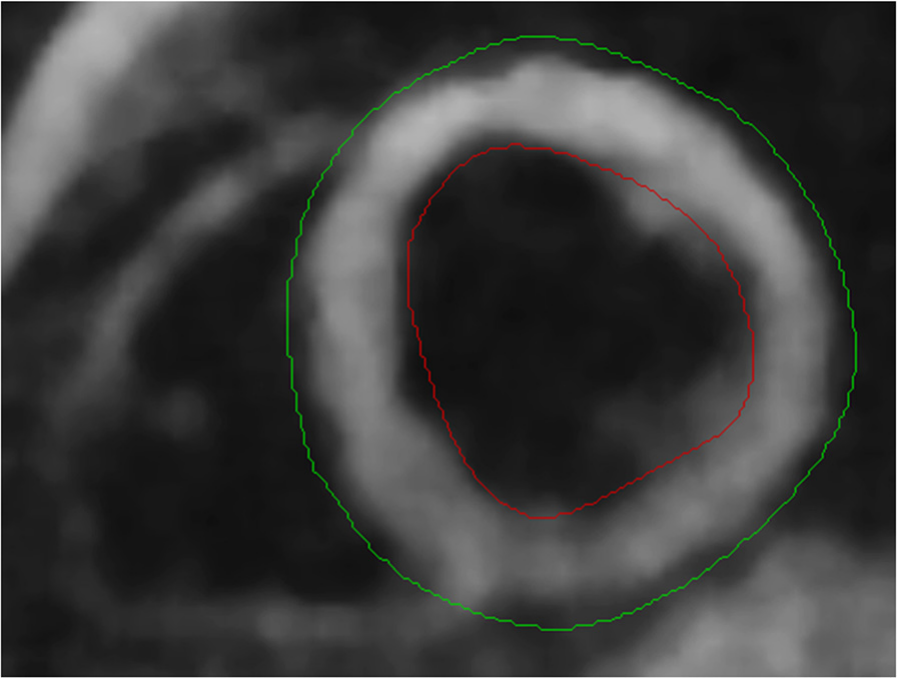
Harmonic magnitude short-axis image the left ventricle with endocardial (red) and epicardial (green) contours.

**Figure 3:**
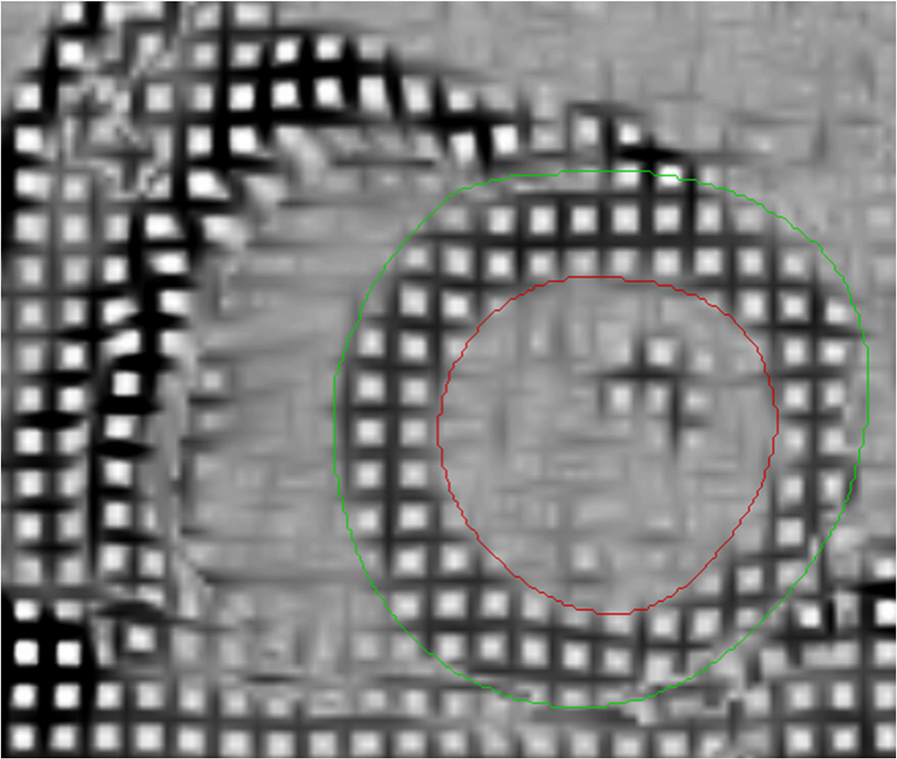
Left ventricle short-axis image with grid of taglines, endocardial (red) and epicardial (green) contours.

CMR images were analyzed offline, using MASS analysis software (Medis, Leiden, The Netherlands). Harmonic magnitude (HARM) and HARP images were computed from the SA CSPAMM images as described by Osman et al. [15]. LV endocardial and epicardial contours were drawn on the HARM images (Figure 2). Myocardial tissue between both contours was tracked by applying the previously described automatic extended HARP tracking method to the HARP images (Figure 3) [16]. Segmental circumferential strain was calculated from Lagrangian strain as a percent change in length of a finite line segment in the circumferential direction. While myocardial fibers of the mid-LV wall are predominantly oriented circumferentially and lie within the short image plane, peak systolic circumferential strain was calculated only from mid-50% of the LV wall. From these segmental circumferential strain datasets, the following parameters were determined: peak systolic CS (peak SCS) and time to peak systolic circumferential strain (T2P-SCS).

### Inter- and intra-observer reliability

The intra-observer variability of CMR-FT was performed in all patients, with a time interval of 2 weeks. The inter-observer reliability of CMR-FT_endo_ and CMR-FT_mid_ was done by 2 experienced, independent observers (L.W. and A.G.) in all 30 patients. In addition, also the intra and inter-observer variability of tissue tagging was determined in 10 patients, who were randomly selected.

### Statistical methods

The IBM SPSS Statistics for Windows, Version 20.0 was used. Continuous variables are expressed in mean ± SD. The intra and inter-observer reliability were assessed using the Intra Class Correlation Coefficient (ICC) with a 2-way random model with absolute agreement. An ICC ≥ 0.70 was considered to be acceptable [17]. Comparison of differences in peak SCS and T2P-SCS between CMR-FT_endo_, tissue tagging and CMR-FT_mid_ was done using the paired t-test, after the data was tested for normal distributions. The related-samples Wilcoxon signed rank test was used when the data was not normally distributed. A p-value of ≤0.05 was considered significant.

## Results

Thirty patients were included. Ten healthy volunteers (mean age 37 ± 11, 9 males, left ventricular ejection fraction (LVEF) 61 ± 6%), 10 patients with LBBB (mean age 62 ± 8 years, 9 males, LVEF 23 ± 7%) and 10 patients with HCM (mean age 53 ± 12 years, 5 males, LVEF 58 ± 8%) were included. Baseline characteristics are presented in Table 1.

**Table 1:** Baseline characteristics

**Baseline characteristics**	**Healthy volunteers n = 10**	**LBBB n = 10**	**HCM n = 10**
Age (yrs)	37 ± 11	62 ± 8	53 ± 12
Male (n)	9 (90%)	9 (90%)	5 (50%)
LVEDV (ml)	180 ± 33	332 ± 89	176 ± 35
LVESV (ml)	69 ± 17	259 ± 87	73 ± 16
LVEF (%)	61 ± 6	23 ± 7	58 ± 8
LV mass (g)	111 ± 28	167 ± 35	164 ± 48

Left bundle branch block patients (LBBB), hypertrophic cardiomyopathy patients (HCM), left ventricular end-diastolic volume (LVEDV), left ventricular end-systolic volume (LVESV), left ventricular ejection fraction (LVEF), left ventricular mass (LV mass).

There were in total 480 segments per analysis method. With CMR-FT all segments were analysable, while with tissue tagging; only 464 segments were analysable.

### Peak systolic circumferential strain

In Table 2, the mean peak SCS for CMR-FT_endo_, tissue tagging and CMR-FT_mid_ are provided. Significant differences are found among these 3 analysis methods regarding the total study group. Mean peak SCS was significantly higher with CMR-FT_endo_ compared with tissue tagging and CMR-FT_mid_. Comparable results were seen in the separate groups between tissue tagging and CMR-FT_mid_, except in the LBBB group. Segmental peak SCS measured with CMR-FT_mid_ and tissue tagging are provided for the healthy volunteers, LBBB and HCM group (Table 3). CMR-FT_mid_ resulted in a higher segmental peak SCS compared with tissue tagging which were most profound in the apical segments.

**Table 2:** **Overview of mean peak SCS and mean T2P-SCS measured with CMR-FT**_**endo**_**, tissue tagging and CMR-FT**_**mid**_

	**Total study group (n = 30)**			**Healthy volunteers (n = 10)**			**LBBB (n = 10)**			**HCM (n = 10)**		
	***CMR-FT***_***endo***_ ***(%)***	***Tissue tagging (%)***	***CMR-FT***_***mid***_ ***(%)***	***CMR-FT***_***endo***_ ***(%)***	***Tissue tagging (%)***	***CMR-FT***_***mid***_ ***(%)***	***CMR-FT***_***endo***_ ***(%)***	***Tissue tagging (%)***	***CMR-FT***_***mid***_ ***(%)***	***CMR-FT***_***endo***_ ***(%)***	***Tissue tagging (%)***	***CMR-FT***_***mid***_ ***(%)***
***Mean peak SCS***	-23.8 ± 9.9^†^	-13.4 ± 3.3	-16.4 ± 6.1^¥$^	-25.9 ± 3.3^#^	-16.5 ± 1.6	-20.0 ± 3.1*^^^	-12.4 ± 5.6	-9.9 ± 1.1	-9.4 ± 3.6^^^	-33.2 ± 5.0^#^	-13.8 ± 2.5	-19.8 ± 3.9^#^^
***Mean T2P-SCS***	380 ± 58	378 ± 52	390 ± 68	336 ± 34*	354 ± 34	330 ± 27*	418 ± 66*	376 ± 55	454 ± 60*	388 ± 41	405 ± 54	384 ± 45

Left bundle branch block patients (LBBB), hypertrophic cardiomyopathy patients (HCM), cardiovascular magnetic resonance myocardial feature-tracking with endocardial contours (CMR-FT_endo_), cardiovascular magnetic resonance myocardial feature-tracking with mid-wall contours (CMR-FT_mid_), systolic circumferential strain (SCS), time to peak systolic circumferential strain (T2P-SCS).

*p < 0.05 compared with tissue tagging; ^#^p < 0.01 compared with tissue tagging; ^^^p < 0.01 compared with CMR-FT_endo_; ^†^p < 0.001 compared with tissue tagging; ^¥^p < 0.001 compared with CMR-FT_endo_; ^$^p = 0.001 compared with tissue tagging.

**Table 3:** Segmental peak SCS, mean ± standard deviation

	**Healthy volunteers (n = 10)**			**LBBB (n = 10)**			**HCM (n = 10)**		
	***CMR-FT***_***mid***_ ***(%)***	***Tissue tagging (%)***	***p-value***	***CMR-FT***_***mid***_ ***(%)***	***Tissue tagging (%)***	***p-value***	***CMR-FT***_***mid***_ ***(%)***	***Tissue tagging (%)***	***p-value***
***1. Basal anterior***	-15.7 ± 9.6	-16.8 ± 2.8	0.58	-9.1 ± 5.8	-9.2 ± 3.5	0.88	-17.6 ± 7.6	-13.9 ± 3.4	0.17
***2. Basal anteroseptal***	-14.2 ± 7.5	-15.6 ± 2.5	0.45	-8.6 ± 9.0	-6.9 ± 3.3	0.51	-11.3 ± 7.5	-13.3 ± 3.9	0.51
***3. Basal septal***	-17.6 ± 3.1	-16.1 ± 2.4	0.17	-5.7 ± 5.4	-7.9 ± 3.9	0.24	-13.6 ± 9.7	-13.3 ± 2.7	0.80
***4. Basal inferior***	-14.5 ± 5.8	-15.4 ± 1.4	0.80	-5.4 ± 4.4	-6.8 ± 3.8	0.51	-20.1 ± 9.0	-14.2 ± 3.8	0.17
***5. Basal posterior***	-18.7 ± 6.3	-19.8 ± 3.0	0.58	-13.1 ± 11.2	-11.6 ± 6.8	0.65	-28.1 ± 11.9	-15.3 ± 2.7	0.02
***6. Basal lateral***	-21.8 ± 7.7	-17.8 ± 3.0	0.09	-13.9 ± 8.1	-13.1 ± 5.7	0.96	-13.8 ± 8.5	-12.5 ± 2.5	0.02
***7. Mid anterior***	-24.2 ± 2.0	-18.1 ± 2.6	0.02	-6.7 ± 3.7	-8.4 ± 3.6	0.39	-26.5 ± 8.9	-16.3 ± 2.3	0.58
***8. Mid anteroseptal***	-15.7 ± 8.2	-16.6 ± 2.0	0.65	-4.8 ± 5.2	-7.4 ± 3.4	0.09	-18.1 ± 10.4	-13.4 ± 5.2	0.17
***9. Mid septal***	-17.3 ± 7.9	-15.7 ± 2.0	0.65	-5.8 ± 5.5	-8.3 ± 4.8	0.17	-15.3 ± 7.7	-13.7 ± 4.7	0.68
***10. Mid inferior***	-22.0 ± 7.8	-15.9 ± 0.9	0.03	-6.3 ± 6.0	-8.0 ± 3.9	0.20	-21.0 ± 12.4	-13.3 ± 4.4	0.09
***11. Mid posterior***	-17.9 ± 9.5	-21.2 ± 2.6	0.65	-7.1 ± 4.3	-12.7 ± 5.6	<0.01	-24.2 ± 10.1	-15.9 ± 2.7	0.09
***12. Mid lateral***	-20.9 ± 6.6	-19.4 ± 2.4	0.65	-10.2 ± 6.4	-13.8 ± 4.0	0.14	-18.0 ± 6.7	-15.5 ± 4.1	0.44
***13. Apical anterior***	-22.7 ± 11.9	-14.4 ± 3.7	<0.01	-11.3 ± 11.1	-9.3 ± 4.7	0.65	-13.7 ± 8.6	-10.7 ± 4.9	0.77
***14. Apical septal***	-17.7 ± 8.7	-12.8 ± 2.6	0.11	-14.6 ± 16.9	-9.1 ± 4.3	0.51	-20.4 ± 9.2	-9.9 ± 3.8	0.03
***15. Apical inferior***	-26.5 ± 8.2	-13.6 ± 3.5	<0.01	-15.1 ± 16.1	-11.4 ± 5.0	0.96	-29.1 ± 9.1	-12.0 ± 4.6	0.01
***16. Apical lateral***	-32.0 ± 4.9	-15.3 ± 1.8	<0.01	-11.7 ± 14.5	-13.7 ± 4.2	0.07	-25.7 ± 10.8	-12.6 ± 5.1	0.01
***Mean***	-20.0 ± 3.1	-16.5 ± 1.6	<0.01	-9.4 ± 3.6	-9.9 ± 1.1	0.67	-19.8 ± 3.9	-13.8 ± 2.5	<0.01

Left bundle branch block patients (LBBB), hypertrophic cardiomyopathy patients (HCM), cardiovascular magnetic resonance myocardial feature-tracking with mid-wall contours (CMR-FT_mid_).

#### Agreement CMR-FT and tissue tagging

In the total study group and the separate groups, the ICC for mean peak SCS between CMR-FT_endo_ and tissue tagging and between CMR-FT_mid_ and tissue tagging was poor (Additional file 1: Table A). The segmental ICC’s are presented in Table 4, showing that in the total study group, 10 of the 16 segments had a significant agreement between CMR-FT_mid_ and tissue tagging, however none of the ICC’s reached an adequate level of 0.70. In the separate groups, no significant agreement was observed in the healthy volunteers group and HCM group, while in the LBBB group only 2 of the 16 segments demonstrates a significant agreement, albeit with an ICC lower than 0.70.

**Table 4:** **Intraclass correlation coefficient of peak SCS between CMR-FT**_**mid **_**and tissue tagging**

	**Total study group (n = 30)**		**Healthy volunteers (n = 10)**		**LBBB (n = 10)**		**HCM (n = 10)**	
	***ICC (95%-CI)***	***p-value***	***ICC (95%-CI)***	***p-value***	***ICC (95%-CI)***	***p-value***	***ICC (95%-CI)***	***p-value***
***1. Basal anterior***	0.41	0.01	0.34	0.17	-0.09	0.60	0.32	0.15
	(0.05-0.67)		(-0.38-0.79)		(-0.78-0.57)		(-0.25-0.78)	
***2. Basal anteroseptal***	0.43	0.01	0.33	0.17	0.47	0.08	0.09	0.40
	(0.05-0.68)		(-0.36-0.78)		(-0.19-0.84)		(-0.57-0.66)	
***3. Basal septal***	0.57	<0.001	-0.21	0.74	0.14	0.33	0.45	0.10
	(0.27-0.77)		(-0.73-0.45)		(-0.47-0.68)		(-0.27-0.83)	
***4. Basal inferior***	0.31	0.04	0.08	0.41	0.35	0.15	-0.36	0.91
	(-0.05-0.60)		(-0.62-0.67)		(-0.31-0.78)		(-0.72-0.29)	
***5. Basal posterior***	0.19	0.14	-0.14	0.64	0.44	0.10	<-0.01	0.51
	(-0.14-.49)		(-0.78-0.54)		(-0.26-0.83)		(-0.24-0.43)	
***6. Basal lateral***	0.32	0.02	0.33	0.12	0.24	0.25	0.15	0.19
	(-0.02-0.60)		(-0.19-0.76)		(-0.50-0.75)		(-0.14-0.63)	
***7. Mid anterior***	0.54	<0.01	0.06	0.39	0.10	0.38	0.05	0.45
	(0.22-0.75)		(-0.26-0.54)		(-0.52-0.66)		(-0.77-0.71)	
***8. Mid anteroseptal***	0.48	<0.01	0.14	0.35	0.18	0.28	0.29	0.19
	(0.13-0.72)		(-0.59-0.70)		(-0.38-0.69)		(-0.31-0.76)	
***9. Mid septal***	0.56	<0.01	0.34	0.16	0.33	0.15	0.36	0.17
	(0.24-0.77)		(-0.36-0.78)		(-0.27-0.77)		(-0.39-0.81)	
***10. Mid inferior***	0.39	0.01	0.12	0.30	0.62	0.02	0.06	0.41
	(0.06-0.65)		(-0.24-0.59)		(0.09-0.89)		(-0.38-0.59)	
***11. Mid posterior***	0.20	0.16	-0.05	0.55	0.33	0.06	-0.16	0.74
	(-0.19-0.53)		(-0.63-0.57)		(-0.14-0.75)		(-0.49-0.43)	
***12. Mid lateral***	0.20	0.15	-0.19	0.70	-0.23	0.77	0.16	0.33
	(-0.19-0.53)		(-0.79-0.49)		(-0.68-0.42)		(-0.50-0.72)	
***13. Apical anterior***	0.300	0.04	0.02	0.48	0.59	0.03	<0.01	0.49
	(-0.04-0.58)		(-0.38-0.54)		(-0.01-0.88)		(-0.65-0.64)	
***14. Apical septal***	0.13	0.20	0.09	0.37	0.13	0.35	0.15	0.22
	(-0.16-0.44)		(-0.38-0.62)		(-0.49-0.67)		(-0.16-0.63)	
***15. Apical inferior***	0.06	0.31	<0.01	0.49	0.05	0.45	6	0.28
	(-0.15-0.33)		(-0.14-0.34)		(-0.62-0.64)		(-0.09-0.44)	
***16. Apical lateral***	0.13	0.19	-0.02	0.78	0.11	0.38	0.25	0.07
	(-0.14-0.42)		(-0.06-0.13)		(-0.60-0.68)		(-0.12-0.73)	
***Mean***	0.58	<0.001	0.19	0.15	0.13	0.36	0.27	<0.01
	(0.14-0.80)		(-0.14-0.62)		(-0.59-0.69)		(-0.07-0.71)	

Intraclass correlation coefficient (ICC), cardiovascular magnetic resonance myocardial feature-tracking with mid-wall contours (CMR-FT_mid_), systolic circumferential strain (SCS), 95%-coincidence interval (95%-CI), left bundle branch block patients (LBBB), hypertrophic cardiomyopathy patients (HCM).

Pearson correlation of the mean peak SCS of the total study group, measured with CMR-FT_mid_ and tissue tagging revealed an R of 0.81 (p < 0.001).

#### Intra and inter-observer variability of CMR-FT and tissue tagging

There was a significantly high intra-observer agreement of mean peak SCS measured with CMR-FT_endo_ and tissue tagging (Additional file 1: Table B). The intra-observer agreement for segmental peak SCS with CMR-FT_mid_ was significant in most segments in the total study group as well as in the LBBB group, this was not present in the same extent in the healthy volunteers group and the HCM group (Additional file 1: Table C). The intra-observer agreement for segmental peak SCS with CMR-FT_mid_ was significantly high in most segments in the total study group. Interestingly, the intra-observer agreement of the apical segments with tissue tagging was lower than the basal and mid segments (Additional file 1: Table D).

The inter-observer agreement of the mean peak SCS was high concerning all 3 analysis methods (Additional file 1: Table B). Segmental peak SCS data for CMR-FT_mid_ is given in the Additional file 1: Table E. The inter-observer agreement of segmental peak SCS in the total study group, measured with CMR-FT_mid_ showed that 10 out of 16 segments yielded an ICC of ≥ 0.70, this was also present in a similar degree in the LBBB group. The mean peak SCS of CMR-FT_mid_ had a significantly high inter-observer agreement in the total study group (ICC 0.93 (95%-CI 0.78-0.97), p < 0.001). Similar result was seen in the LBBB group, whereas in the healthy volunteers group and HCM group the ICC was lower. Segmental peak SCS data of tissue tagging showed that 11 out of 16 segments had an ICC ≥ 0.70 (Additional file 1: Table D).

### Time to peak systolic circumferential strain

In Table 2, the mean T2P-SCS for CMR-FT_endo_, tissue tagging and CMR-FT_mid_ are provided. In the total study group, no significant differences were seen among the 3 analysis methods. In the healthy volunteers and LBBB group, significant differences were observed in CMR-FT_endo_ and CMR-FT_mid_, compared with tissue tagging.

Segmental T2P-SCS values of CMR-FT_mid_ and tissue tagging are presented in Table 5. Importantly, T2P-SCS of the septal segments was significantly longer in the LBBB group when measured with CMR-FT_mid_ compared with tissue tagging (basal anteroseptum: 430 ± 179 ms versus 226 ± 194 ms, p = 0.04, respectively, mid anteroseptal: 552 ± 232 ms versus 147 ± 153 ms, p < 0.01, respectively). In contrast, T2P-SCS of the basal posterior segments was significantly shorter in the LBBB group when measuredwith CMR-FT_mid_ compared with tissue tagging (476 ± 138 ms versus 530 ± 186 ms, p = 0.04, respectively). These 2 findings combined might largely underestimate the extent of left ventricular dyssynchrony of the basal LV slice in this particular patient group compared with tissue tagging. Additional analysis showed that in this patient group, the basal septal segments measured with CMR-FT_mid_ gives a significant longer T2P-SCS compared with tissue tagging (419 ± 157 ms versus 253 ± 176 ms, p = 0.01, respectively), while the basal lateral segments measured with CMR-FT_mid_ were not significantly different compared with tissue tagging 438 ± 74 ms versus 486 ± 115 ms, p = 0.09, respectively.

**Table 5:** Segmental T2P-SCS, mean ± standard deviation

	**Healthy volunteers** ***(n = 10)***			**LBBB** ***(n = 10)***			**HCM (n = 10)**		
	***CMR-FT***_***mid***_ ***(ms)***	***Tissue tagging (ms)***	***p-value***	***CMR-FT***_***mid***_ ***(ms)***	***Tissue tagging (ms)***	***p-value***	***CMR-FT***_***mid***_ ***(ms)***	***Tissue tagging (ms)***	***p-value***
***1. Basal anterior***	324 ± 94	301 ± 38	0.80	411 ± 157	350 ± 113	0.72	377 ± 122	373 ± 75	0.77
***2. Basal anteroseptal***	324 ± 125	307 ± 57	0.72	430 ± 179	226 ± 194	0.04	457 ± 219	402 ± 74	0.51
***3. Basal septal***	288 ± 27	311 ± 36	0.20	408 ± 250	280 ± 186	0.13	355 ± 157	344 ± 58	0.80
***4. Basal inferior***	313 ± 93	354 ± 45	0.33	536 ± 163	429 ± 105	0.09	332 ± 68	377 ± 130	0.45
***5. Basal posterior***	331 ± 29	403 ± 40	<0.01	476 ± 138	530 ± 186	0.04	462 ± 151	358 ± 110	0.01
***6. Basal lateral***	321 ± 48	332 ± 54	0.80	399 ± 44	443 ± 66	0.06	420 ± 97	400 ± 69	0.21
***7. Mid anterior***	317 ± 43	305 ± 29	0.29	458 ± 192	393 ± 155	0.65	288 ± 169	493 ± 77	0.03
***8. Mid anteroseptal***	327 ± 144	314 ± 53	0.65	552 ± 232	147 ± 153	<0.01	442 ± 196	418 ± 90	0.68
***9. Mid septal***	355 ± 55	338 ± 59	0.72	364 ± 233	358 ± 251	0.65	384 ± 181	404 ± 120	0.95
***10. Mid inferior***	339 ± 54	365 ± 60	0.33	418 ± 233	448 ± 115	0.96	402 ± 177	398 ± 92	0.39
***11. Mid posterior***	323 ± 49	392 ± 36	<0.01	511 ± 139	468 ± 85	0.65	399 ± 57	403 ± 63	0.59
***12. Mid lateral***	328 ± 43	351 ± 42	0.33	461 ± 82	430 ± 80	0.72	420 ± 99	417 ± 106	0.95
***13. Apical anterior***	367 ± 63	379 ± 61	0.72	560 ± 171	384 ± 163	0.05	339 ± 138	483 ± 99	0.02
***14. Apical septal***	352 ± 147	408 ± 48	0.14	354 ± 201	330 ± 147	0.88	365 ± 110	414 ± 150	0.33
***15. Apical inferior***	327 ± 41	402 ± 66	<0.01	454 ± 117	380 ± 92	0.07	340 ± 82	463 ± 119	0.02
***16. Apical lateral***	350 ± 45	405 ± 45	0.04	476 ± 114	428 ± 85	0.20	420 ± 101	425 ± 156	1.00
***Mean***	330 ± 27	354 ± 34	0.02	454 ± 60	376 ± 56	<0.01	384 ± 45	404 ± 54	0.17

Time to peak systolic circumferential strain (T2P-SCS), left bundle branch block patients (LBBB), hypertrophic cardiomyopathy patients (HCM), cardiovascular magnetic resonance myocardial feature-tracking with mid-wall contours (CMR-FT_mid_).

#### Agreement of CMR-FT and tissue tagging

The ICC’s for mean T2P-SCS between CMR-FT_endo_, tissue tagging and CMR-FT_mid_ are given in the Additional file 1: Table A. In the total study group, the ICC of CMR-FT_endo_ and tissue tagging did not reach an adequate level of 0.70, whereas in the separated groups the healthy volunteers and HCM group reached an adequate level of 0.70. Further, the ICC of CMR-FT_mid_ was low for the total study group and none of the separate groups showed an ICC ≥ 0.70. Segmental ICC’s between CMR-FT_mid_ and tissue tagging revealed low ICC’s (Table 6). A Pearson correlation of the mean T2P-SCS was performed in the total study group, an R = 0.40 (p = 0.03) was found between CMR-FT_mid_ and tissue tagging.

**Table 6:** **Intraclass correlation coefficient of T2P-SCS between CMR-FT**_**mid **_**and tissue tagging**

	**Total study group (n = 30)**		**Healthy volunteers (n = 10)**		**LBBB (n = 10)**		**HCM (n = 10)**	
	***ICC (95%-CI)***	***p-value***	***ICC (95%-CI)***	***p-value***	***ICC (95%-CI)***	***p-value***	***ICC (95%-CI)***	***p-value***
***1. Basal anterior***	0.10	0.29	0.34	0.16	-0.21	0.73	0.39	0.15
	(-0.26-0.45)		(-0.32-0.78)		(-0.76-0.46)		(-0.41-0.83)	
***2. Basal anteroseptal***	0.19	0.12	0.31	0.19	0.17	0.22	0.14	0.35
	(-0.12-0.49)		(-0.41-0.78)		(-0.20-0.63)		(-0.53-0.69)	
***3. Basal septal***	0.28	0.06	-0.24	0.79	0.35	0.12	0.23	0.27
	(-0.07-0.58)		(-0.67-0.40)		(-0.21-0.77)		(-0.52-0.74)	
***4. Basal inferior***	0.36	0.03	0.36	0.11	0.27	0.16	<0.01	0.49
	(<-0.01-0.64)		(-0.20-0.78)		(-0.22-0.72)		(-0.61-0.61)	
***5. Basal posterior***	0.72	<0.001	0.24	<0.01	0.86	<0.001	0.60	<0.01
	(0.49-0.86)		(-0.05-0.69)		(0.45-0.97)		(-0.08-0.89)	
***6. Basal lateral***	0.48	<0.01	0.26	0.23	-0.55	0.98	0.75	<0.01
	(0.14-0.71)		(-0.45-0.75)		(-0.85-0.15)		(0.26-0.94)	
***7. Mid anterior***	<0.01	0.48	0.47	0.07	0.12	0.37	-0.05	0.60
	(-0.37-0.38)		(-0.15-0.84)		(-0.53-0.67)		(-0.28-0.45)	
***8. Mid anteroseptal***	-0.08	0.70	0.36	0.15	-0.05	0.66	0.59	0.04
	(-0.33-0.24)		(-0.37-0.80)		(-0.20-0.31)		(-0.09-0.89)	
***9. Mid septal***	-0.08	0.66	-0.17	0.68	-0.14	0.64	0.02	0.48
	(-0.45-0.30)		(-0.78-0.51)		(-0.81-0.54)		(-0.75-0.67)	
***10. Mid inferior***	0.08	0.33	0.06	0.43	-0.01	0.51	0.08	0.41
	(-0.29-0.43)		(-0.55-0.64)		(-0.71-0.62)		(-0.65-0.67)	
***11. Mid posterior***	0.22	0.13	0.22	0.07	-0.21	0.72	<-0.01	0.51
	(-0.16-0.54)		(-0.12-0.65)		(-0.78-0.48)		(-0.77-0.65)	
***12. Mid lateral***	0.35	0.03	-0.04	0.55	0.05	0.44	0.17	0.34
	(-0.02-0.64)		(-0.60-0.56)		(-0.59-0.64)		(-0.64-0.74)	
***13. Apical anterior***	-0.13	0.74	0.08	0.41	-0.09	0.65	0.18	0.20
	(-0.49-0.26)		(-0.63-0.67)		(-0.44-0.45)		(-0.18-0.65)	
***14. Apical septal***	0.20	0.16	-0.10	0.62	0.32	0.19	0.24	0.27
	(-0.19-0.53)		(-0.66-0.53)		(-0.41-0.78)		(-0.52-0.78)	
***15. Apical inferior***	0.28	0.06	0.33	0.02	0.39	0.07	0.39	0.03
	(-0.08-0.58)		(-0.12-0.76)		(-0.14-0.79)		(-0.13-0.82)	
***16. Apical lateral***	0.50	<0.01	0.08	0.35	0.50	0.05	0.60	0.06
	(0.16-0.74)		(-0.23-0.54)		(-0.07-0.84)		(-0.18-0.91)	
***Mean***	0.38	0.02	0.49	0.02	0.17	0.18	0.60	0.02
	(0.04-0.65)		(-0.08-0.84)		(-0.15-0.61)		(0.06-0.88)	

Intraclass correlation coefficient (ICC), time to systolic circumferential strain (T2P-SCS), cardiovascular magnetic resonance myocardial feature-tracking with mid-wall contours (CMR-FT_mid_), 95%-coincidence interval (95%-CI), left bundle branch block patients (LBBB), hypertrophic cardiomyopathy patients (HCM).

#### Intra and inter-observer variability of CMR-FT and tissue tagging

All 3 analysis methods show a high significant intra-observer agreement of T2P-SCS (Additional file 1: Table B). However, the intra-observer agreement of mean T2P-SCS measured with CMR-FT_endo_ seems to be higher than the intra-observer agreement measured with CMR-FT_mid_. Segmental analysis revealed that in the total study group, 11 segments have a significant intra-observer agreement of T2P-SCS with CMR-FT_mid_ (Additional file 1: Table F). In the healthy volunteers group 10 segments; in the LBBB group 7 segments and in the HCM group 5 segments showed a significant agreement. In addition, tissue tagging showed the highest intra-observer agreement concerning the segmental ICC’s of T2P-SCS (Additional file 1: Table C). The inter-observer agreement is high in CMR-FT_endo_ and CMR-FT_mid_, in contrast to tissue tagging (Additional file 1: Table B). The inter-observer agreement of the segmental T2P-SCS of CMR-FT_mid_ is given in the Additional file 1: Table G. A significant agreement of segmental T2P-SCS with CMR-FT_mid_ was found for 13 segments in the total study group, in the healthy volunteers group, 10 segments; in the LBBB group, 7 segments and in the HCM group, 5 segments. Segmental T2P-SCS analysis with tissue tagging showed that a significant agreement was found in only 7 out of 16 segments with only a few segments with an ICC over the 0.70, resulting in an overall low inter-observer agreement.

## Discussion

The present study was conducted to validate segmental peak circumferential strain and time to peak circumferential strain analysed by CMR-FT with tissue tagging, and to determine its intra and inter-observer reliability in healthy volunteers, patients with left bundle branch block and in patients with hypertrophic obstructive cardiomyopathy.

CMR-FT_endo_ provides the highest peak SCS and tissue tagging the lowest, CMR-FT_mid_ provides peak SCS values between the values of CMR-FT_endo_ and tissue tagging. However, differences in peak SCS might be explained by the different calculation methods. CMR-FT calculates the motion of a tissue voxel, while tissue tagging quantifies tag deformation of a myocardial segment of several millimetres. Since myocardial fibers allocated in the mid-wall are mostly circumferentially orientated and tissue tagging analyses the mid-wall of the cardiac wall, we decided to track the mid-wall with CMR-FT in order to make an accurate comparison. The myocardial fibers allocated in the endocardium are more radially orientated, this might explain the differences in peak SCS measured with CMR-FT_endo_ and tissue tagging and CMRF-FT_mid_. Further, it is important to focus on segmental strain, since knowledge of the mechanism of segmental LV dyssynchrony is of interest in many cardiac diseases [18-21]. No significant differences in mean T2P-SCS were found in the total study group between the 3 analysis methods, but differences were present in the healthy volunteers and LBBB group.

Because differences in absolute peak SCS and T2P-SCS are seen in several segments analysed with CMR-FT_mid_ compared with tissue tagging, CMR-FT_mid_ appears not to be useful in cardiac diseases where it is important to evaluate segmental strain.

### Agreement CMR-FT and tissue tagging

Limited studies have been conducted to investigate the segmental strain measured with CMR-FT compared with tissue tagging [22]. Hor et al. [11] found there is a strong correlation between mean strain measured with CMR-FT and tissue tagging, confirmed by our data. However, they disregarded the valuable information of segmental circumferential strain. Harrild et al. [22] performed a validation study with segmental peak circumferential strain and segmental time to peak circumferential strain in normal subjects and patients with HCM, measured with CMR-FT compared with tissue tagging. They concluded that there is a modest segmental peak circumferential strain agreement, which is better in normal subjects and the segmental time to peak circumferential strain agreement is also better in normal subjects than in HCM patients. Our data could not confirm this observation. This might be explained by the fact that Harrild et al. performed CMR-FT at the endocardial border of the LV, while we performed CMR-FT at the level of the mid-wall of the LV.

### Intra and inter-observer variability of CMR-FT and tissue tagging

The intra-observer agreement of the mean peak SCS was good in CMR-FT_endo_ and tissue tagging. In CMR-FT_mid_, the intra-observer agreement of mean peak SCS is low, while the intra-observer agreement of the healthy volunteers group and LBBB group is good. The intra-observer agreement in the HCM group varies; some segments show no agreement at all, while in other segments a very low agreement is seen. An explanation can be that HCM patients often have asymmetric hypertrophy, which makes it difficult to draw the mid-wall contours correctly.

The inter-observer agreement of the segmental peak SCS of tissue tagging seemed to be better than CMR-FT_mid_. The mean peak SCS shows there is a strong intra and inter-observer reproducibility, which seems to be better with CMR-FT_endo_ than with CMR-FT_mid_. Earlier studies demonstrated that there is a strong intra and inter-observer reproducibility of CMR-FT [11]. Although Schuster et al. [23] evaluated the intra-observer reproducibility of CMR-FT at 1.5 T and 3 T CMR, they concluded that the intra-observer reproducibility of peak SCS was better on a global rather than on a segmental level concerning peak SCS. The intra and inter-observer agreement of tissue tagging to calculate segmental peak SCS is better than with CMR-FT_mid_, since there is an excellent agreement in most of the segments.

The intra-observer agreement of T2P-SCS is good, regarding all 3 analysis methods. However, tissue tagging has the best intra-observer agreement of T2P-SCS compared with CMR-FT_endo_ and CMR-FT_mid_. The intra-observer agreement of segmental T2P-SCS measured with CMR-FT_mid_ is subject to variation, while the intra and inter-observer reproducibility of the mean T2P-SCS is less subject to variation. The inter-observer agreement of segmental T2P-SCS with tissue tagging is also subject to variation, but with tissue tagging there are more segments with a higher ICC, when compared with CMR-FT_mid_. Therefore, tissue tagging is still considered as the gold standard for strain analysis for research purposes.

### Limitations

The present study contains a small number of patients, especially the separate patient groups. Further, the slice levels for CMR-FT and tissue tagging were similar but not identical, since the images for tissue tagging is not always planned on exactly the same levels as CMR-FT. Also, the contours used for the CMR-FT_mid_ are drawn on the mid-wall of the left ventricle and therefore it is more subjected to variation. While for tissue tagging, the endo- and epicardial contours were drawn and the mid-wall was calculated.

## Conclusion

In the present study population, absolute values of peak SCS are higher with CMR-FT_endo_ compared with tissue tagging and CMR-FT_mid_. Differences in T2P-SCS are also present between the 3 analysis methods. The intra and inter-observer agreement of segmental peak SCS, as well as T2P-SCS is substantially lower with CMR-FT_mid_ compared with tissue tagging. Therefore, current segmental CMR-FT_mid_ techniques are not yet applicable for clinical and research purposes.

## Abbreviations

95%-CI: 95%-confidence interval; CMR: Cardiovascular magnetic resonance; CMR-FT: Cardiovascular magnetic resonance myocardial feature tracking; CSPAMM: Complementary spatial modulation of magnetization; HARM: Harmonic magnitude; HARP: Harmonic phase; HCM: Hypertrophic cardiomyopathy; ICC: Intra class correlation coefficient; LBBB: Left bundle branch block; LV: Left ventricular; LVEF: Left ventricular ejection fraction; Peak SCS: Peak systolic circumferential strain; SA: Short-axis; SSFP: Steady-state free precession; T2P-SCS: Time to peak systolic circumferential strain.

## Competing interests

The authors declare that they have no competing interests.

## Authors’ contributions

LW was involved in the acquisition, analysis and interpretation the data and drafted the manuscript. TG was involved in the interpretation of the data, the conception and design of the manuscript, revised the manuscript and has given the final approval of the version to be published. AG was involved in the acquisition and analysis of the data and revised the manuscript critically. MWH was involved in the analysis of the data and revised the manuscript critically. CPA was involved in the design of the manuscript and revised the manuscript critically. ACR was involved in the design of the manuscript, revised the manuscript critically and has given the final approval of the version to be published. All authors read and approved the final manuscript.

## Supplementary Material

Additional file 1 **Table A: ICC between CMR-FT**_**endo**_** and tissue tagging; and between CMR-FT**_**mid**_** and tissue tagging.** Table B: Intra and inter-observer variability of peak SCS and T2P-SCS of CMR-FT_endo_, tissue tagging and CMR-FT_mid_. Table C: Intra-observer variability CMR-FT_mid_, ICC of peak SCS. Table D: Intra- and inter observer variability tissue tagging, ICC of peak SCS and T2P-SCS. Table E: Inter-observer variability of CMR-FT_mid_, ICC of peak SCS. Table F: Intra-observer variability CMR-FT_mid_, ICC of T2P-SCS. Table G: Inter-observer variability of CMR-FT_mid_, ICC of T2P-SCS. Click here for file
