# Supplementary material for: Feature tracking compared with tissue tagging measurements of segmental strain by cardiovascular magnetic resonance
Source: J Cardiovasc Magn Reson. 2014 Jan 22;16(1):10. doi: 10.1186/1532-429X-16-10 (PMC3926943; doi:10.1186/1532-429X-16-10)
Supplement: Additional file 1 — Table A: ICC between CMR-FT endo and tissue tagging; and between CMR-FT mid and tissue tagging. Table B: Intra and inter-observer variability of peak SCS and T2P-SCS of CMR-FTendo, tissue tagging and CMR-FTmid. Table C: Intra-observer variability CMR-FTmid, ICC of peak SCS. Table D: Intra- and inter observer variability tissue tagging, ICC of peak SCS and T2P-SCS. Table E: Inter-observer variability of CMR-FTmid, ICC of peak SCS. Table F: Intra-observer variability CMR-FTmid, ICC of T2P-SCS. Table G: Inter-observer variability of CMR-FTmid, ICC of T2P-SCS. [file 1532-429X-16-10-S1.docx]

Additional file 1. Table A: ICC between CMR-FT_endo_ and tissue tagging; and between CMR-FT_mid_ and tissue tagging

|  | **Total study group**  **(n = 30)** | | **Healthy volunteers (n = 10)** | | **LBBB (n = 10)** | | **HCM (n = 10)** | |
| --- | --- | --- | --- | --- | --- | --- | --- | --- |
|  | ***ICC***  ***(95%-CI)*** | ***p-value*** | ***ICC***  ***(95%-CI)*** | ***p-value*** | ***ICC***  ***(95%-CI)*** | ***p-value*** | ***ICC***  ***(95%-CI)*** | ***p-value*** |
| ***Mean peak SCS CMR-FT_endo_*** | 0.19  (-0.10-0.49) | 0.02 | 0.03  (-0.04-0.23) | 0.26 | 0.15  (-0.38-0.67) | 0.31 | 0.03  (-0.02-0.19) | 0.16 |
| ***Mean peak SCS CMR-FT_mid_*** | 0.58  (0.14-0.80) | <0.001 | 0.19  (-0.14-0.62) | 0.15 | 0.13  (-0.59-0.69) | 0.36 | 0.27  (-0.07-0.71) | <0.01 |
| ***Mean T2P-SCS CMR-FT_endo_*** | 0.64  (0.36-0.81) | <0.001 | 0.78  (0.05-0.95) | <0.001 | 0.49  (-0.07-0.84) | 0.03 | 0.70  (0.21-0.91) | <0.01 |
| ***Mean T2P-SCS CMR-FT_mid_*** | 0.38  (0.04-0.65) | 0.02 | 0.49  (-0.08-0.84) | 0.02 | 0.17  (-0.15-0.61) | 0.18 | 0.60  (0.06-0.88) | 0.02 |

Intraclass correlation coefficient (ICC), 95%-coincidence interval (95%-CI), left bundle branch block patients (LBBB), hypertrophic cardiomyopathy patients (HCM), cardiovascular magnetic resonance imaging myocardial feature-tracking with endocardial contours (CMR-FT_endo_), cardiovascular magnetic resonance imaging myocardial feature-tracking with mid-wall contours (CMR-FT_mid_)

Additional file 1. Table B: Intra and inter-observer variability of peak SCS and T2P-SCS of CMR-FT_endo_, tissue tagging and CMR-FT_mid_

|  | **CMR-FT_endo_**  **(n = 30)** | | **Tissue tagging**  **(n = 10)** | | **CMR-FT_mid_**  **(n = 30)** | |
| --- | --- | --- | --- | --- | --- | --- |
|  | ***ICC***  ***(95%-CI)*** | ***p-value*** | ***ICC***  ***(95%-CI)*** | ***p-value*** | ***ICC***  ***(95%-CI)*** | ***p-value*** |
| ***Intra-observer variability mean peak SCS*** | 0.89  (0.21-0.97) | <0.001 | 0.99  (0.93-1.00) | <0.001 | 0.59  (0.21-0.80) | <0.001 |
| ***Inter-observer variability mean peak SCS*** | 0.99  (0.98-1.00) | <0.001 | 0.83  (0.20-0.96) | <0.001 | 0.93  (0.78-0.97) | <0.001 |
| ***Intra-observer variability mean T2P-SCS*** | 0.84  (0.70-0.92) | <0.001 | 0.98  (0.91-0.99) | <0.001 | 0.75  (0.54-0.87) | <0.001 |
| ***Inter-observer variability mean T2P-SCS*** | 0.88  (0.77-0.94) | <0.001 | 0.32  (-0.42-0.79) | 0.19 | 0.77  (0.57-0.88) | <0.001 |

Intraclass correlation coefficient (ICC), 95%-coincidence interval (95%-CI), cardiovascular magnetic resonance imaging myocardial feature-tracking with endocardial contours (CMR-FT_endo_), cardiovascular magnetic resonance imaging myocardial feature-tracking with mid-wall contours (CMR-FT_mid_), systolic circumferential strain (SCS), time to peak systolic circumferential strain (T2P-SCS)

Additional file 1. Table C: Intra-observer variability CMR-FT_mid_, ICC of peak SCS

|  | **Total study group**  **(n = 30)** | | **Healthy volunteers (n = 10)** | | **LBBB (n = 10)** | | **HCM (n = 10)** | |
| --- | --- | --- | --- | --- | --- | --- | --- | --- |
|  | ***ICC***  ***(95%-CI)*** | ***p-value*** | ***ICC***  ***(95%-CI)*** | ***p-value*** | ***ICC***  ***(95%-CI)*** | ***p-value*** | ***ICC***  ***(95%-CI)*** | ***p-value*** |
| ***1. basal anterior*** | 0.45  (0.12-0.69) | <0.01 | 0.56  (-0.08-0.87) | 0.04 | 0.73  (0.26-0.92) | <0.01 | 0.03  (-0.19-0.45) | 0.42 |
| ***2. basal anteroseptal*** | 0.35  (0.02-0.62) | 0.02 | 0.92  (0.37-0.98) | <0.001 | 0.77  (0.34-0.94) | <0.01 | 0.12  (-0.04-0.50) | 0.02 |
| ***3. basal septal*** | 0.53  (0.21-0.75) | <0.001 | 0.28  (-0.27-0.74) | 0.17 | 0.79  (0.35-0.94) | <0.01 | 0.31  (-0.09-0.74) | 0.01 |
| ***4. basal inferior*** | 0.70  (0.44-0.85) | <0.001 | 0.59  (-0.06-0.88) | 0.04 | 0.36  (-0.37-0.80) | 0.16 | 0.32  (-0.13-0.75) | 0.03 |
| ***5. basal posterior*** | 0.67  (0.41-0.83) | <0.001 | 0.21  (-0.41-0.72) | 0.26 | 0.88  (0.62-0.97) | <0.001 | 0.33  (-0.15-0.75) | 0.09 |
| ***6. basal lateral*** | 0.60  (0.31-0.79) | <0.001 | 0.64  (0.02-0.90) | 0.02 | 0.65  (0.06-0.90) | 0.02 | 0.19  (-0.44-0.71) | 0.28 |
| ***7. mid anterior*** | 0.33  (<0.01-0.61) | 0.02 | 0.22  (-0.51-0.74) | 0.27 | 0.57  (-0.11-0.87) | 0.05 | <-0.01  (-0.21-0.40) | 0.52 |
| ***8. mid anteroseptal*** | 0.51  (0.16-0.74) | <0.001 | 0.89  (0.63-0.97) | <0.001 | 0.66  (0.08-0.90) | 0.02 | 0.10  (-0.12-0.51) | 0.23 |
| ***9. mid septal*** | 0.47  (0.03-0.74) | <0.001 | 0.83  (0.26-0.96) | <0.001 | 0.77  (0.32-0.94) | <0.01 | 0.13  (-0.03-0.52) | <0.01 |
| ***10. mid inferior*** | 0.55  (0.25-0.75) | <0.001 | 0.41  (-0.33-0.82) | 0.13 | 0.73  (0.20-0.93) | <0.01 | 0.05  (-0.28-0.54) | 0.41 |
| ***11. mid posterior*** | 0.70  (0.44-0.85) | <0.001 | 0.86  (0.54-0.96) | <0.001 | 0.55  (-0.09-0.87) | 0.05 | 0.10  (-0.18-0.54) | 0.29 |
| ***12. mid lateral*** | 0.51  (0.20-0.73) | <0.001 | 0.37  (-0.30-0.79) | 0.14 | 0.77  (0.35-0.94) | <0.01 | 0.16  (-0.24-0.63) | 0.26 |
| ***13. apical anterior*** | 0.31  (-0.01-0.59) | 0.03 | 0.09  (-0.65-0.68) | 0.40 | 0.95  (0.82-0.99) | <0.001 | 0.08  (-0.08-0.42) | 0.19 |
| ***14. apical septal*** | 0.60  (0.20-0.81) | <0.001 | 0.82  (0.47-0.95) | 0.001 | 0.96  (0.86-0.99) | <0.001 | -0.01  (-0.10-0.23) | 0.57 |
| ***15. apical inferior*** | 0.73  (0.51-0.86) | <0.001 | 0.82  (0.43-0.95) | 0.001 | 0.97  (0.87-0.99) | <0.001 | -0.15  (-0.44-0.37) | 0.78 |
| ***16. apical lateral*** | 0.70  (0.46-0.85) | <0.001 | 0.29  (-0.27-0.75) | 0.17 | 0.94  (0.79-0.99) | <0.001 | 0.06  (-0.35-0.58) | 0.41 |
| ***Mean*** | 0.59  (0.21-0.80) | <0.001 | 0.87  (0.57-0.97) | <0.001 | 0.96  (0.83-0.99) | <0.001 | 0.09  (-0.03-0.40) | 0.02 |

Cardiovascular magnetic resonance imaging myocardial feature-tracking with mid-wall contours (CMR-FT_mid_), intraclass correlation coefficient (ICC), systolic circumferential strain (SCS), 95%-confidence interval (95%-CI), left bundle branch block patients (LBBB), hypertrophic cardiomyopathy patients (HCM)

Additional file 1. Table D: Intra- and inter observer variability tissue tagging, ICC of peak SCS and T2P-SCS

|  | **Intra-observer variability peak SCS**  **Total study group (n = 10)** | | **Inter-observer variability peak SCS**  **Total study group (n = 10)** | | **Intra-observer variability T2P-SCS**  **Total study group (n = 10)** | | **Inter-observer variability T2P-SCS**  **Total study group (n = 10)** | |
| --- | --- | --- | --- | --- | --- | --- | --- | --- |
|  | ***ICC***  ***(95%-CI)*** | ***p-value*** | ***ICC***  ***(95%-CI)*** | ***p-value*** | ***ICC***  ***(95%-CI)*** | ***p-value*** | ***ICC***  ***(95%-CI)*** | ***p-value*** |
| ***1. basal anterior*** | 0.89  (0.62-0.97) | <0.001 | 0.84  (0.47-0.96) | 0.001 | 0.52  (-0.14-0.86) | 0.06 | 0.48  (-0.12-0.84) | 0.06 |
| ***2. basal anteroseptal*** | 0.97  (0.89-0.99) | <0.001 | 0.94  (0.76-0.99) | <0.001 | 1.00  (0.97-1.00) | <0.001 | 0.47  (-0.14-0.83) | 0.07 |
| ***3. basal septal*** | 0.98  (0.91-0.99) | <0.001 | 0.86  (0.54-0.96) | <0.001 | 0.97  (0.89-0.99) | <0.001 | -0.02  (-0.69-0.61) | 0.52 |
| ***4. basal inferior*** | 0.91  (0.69-0.98) | <0.001 | 0.46  (-0.11-0.83) | 0.06 | 0.92  (0.72-0.98) | <0.001 | 0.53  (-0.11-0.86) | 0.05 |
| ***5. basal posterior*** | 0.89  (0.64-0.98) | <0.001 | 0.88  (0.55-0.97) | <0.001 | 0.94  (0.79-0.99) | <0.001 | -0.29  (-0.84-0.42) | 0.79 |
| ***6. basal lateral*** | 0.98  (0.92-0.99) | <0.001 | 0.87  (0.46-0.97) | <0.001 | 0.81  (0.42-0.95) | 0.001 | 0.83  (0.45-0.95) | 0.001 |
| ***7. mid anterior*** | 0.97  (0.89-0.99) | <0.001 | 0.89  (0.64-0.97) | <0.001 | 0.93  (0.74-0.98) | <0.001 | 0.87  (0.56-0.97) | <0.001 |
| ***8. mid anteroseptal*** | 0.94  (0.73-0.99) | <0.001 | 0.86  (0.55-0.96) | <0.001 | 0.80  (0.41-0.95) | <0.01 | 0.53  (-0.07-0.86) | 0.05 |
| ***9. mid septal*** | 0.88  (0.61-0.97) | <0.001 | 0.85  (0.52-0.96) | 0.001 | 0.98  (0.91-0.99) | <0.001 | 0.07  (-0.67-0.66) | 0.43 |
| ***10. mid inferior*** | 0.83  (0.47-0.95) | 0.001 | 0.82  (0.42-0.95) | 0.001 | 0.52  (-0.07-0.85) | 0.05 | 0.27  (-0.48-0.76) | 0.23 |
| ***11. mid posterior*** | 0.97  (0.88-0.99) | <0.001 | 0.94  (0.78-0.98) | <0.001 | 0.57  (-0.10-0.88) | 0.04 | 0.77  (0.30-0.94) | <0.01 |
| ***12. mid lateral*** | 0.89  (0.17-0.98) | <0.001 | 0.79  (0.38-0.94) | 0.001 | 0.75  (0.27-0.93) | <0.01 | 0.85  (0.50-0.96) | 0.001 |
| ***13. apical anterior*** | 0.83  (0.47-0.96) | 0.001 | 0.49  (-0.08-0.84) | 0.03 | 0.36  (-0.36-0.80) | 0.15 | 0.42  (-0.29-0.82) | 0.11 |
| ***14. apical septal*** | 0.87  (0.55-0.97) | <0.001 | 0.51  (-0.05-0.84) | 0.03 | 0.90  (0.63-0.97) | <0.001 | 0.21  (-0.50-0.73) | 0.28 |
| ***15. apical inferior*** | 0.71  (0.18-0.92) | <0.01 | -0.03  (-0.65-0.61) | 0.54 | 0.66  (0.11-0.90) | 0.02 | -0.31  (-0.89-0.44) | 0.79 |
| ***16. apical lateral*** | 0.70  (0.19-0.91) | <0.01 | 0.27  (-0.27-0.73) | 0.18 | 0.61  (<-0.01-0.89) | 0.03 | 0.62  (0.09-0.89) | 0.01 |
| ***Mean*** | 0.99  (0.93-1.00) | <0.001 | 0.83  (0.20-0.96) | <0.001 | 0.98  (0.91-0.99) | <0.001 | 0.32  (-0.42-0.78) | 0.19 |

Intraclass correlation coefficient (ICC), systolic circumferential strain (SCS), time to peak systolic circumferential strain (T2P-SCS), 95%-confidence interval (95%-CI), left bundle branch block patients (LBBB), hypertrophic cardiomyopathy patients (HCM)

Additional file 1. Table E: Inter-observer variability of CMR-FT_mid_, ICC of peak SCS

|  | **Total study group**  **(n = 30)** | | **Healthy volunteers (n = 10)** | | **LBBB (n = 10)** | | **HCM (n = 10)** | |
| --- | --- | --- | --- | --- | --- | --- | --- | --- |
|  | ***ICC***  ***(95%-CI)*** | ***p-value*** | ***ICC***  ***(95%-CI)*** | ***p-value*** | ***ICC***  ***(95%-CI)*** | ***p-value*** | ***ICC***  ***(95%-CI)*** | ***p-value*** |
| ***1. basal anterior*** | 0.52  (0.20-0.74) | 0.001 | 0.32  (-0.23-0.76) | 0.13 | 0.60  (-0.04-0.89) | 0.03 | 0.31  (-0.18-0.74) | 0.11 |
| ***2. basal anteroseptal*** | 0.56  (0.25-0.76) | 0.001 | 0.49  (-0.12-0.84) | 0.06 | 0.66  (0.13-0.90) | <0.01 | 0.37  (-0.22-0.78) | 0.12 |
| ***3. basal septal*** | 0.72  (0.49-0.86) | <0.001 | 0.12  (-0.36-0.63) | 0.34 | 0.61  (-0.01-0.89) | 0.03 | 0.78  (0.34-0.94) | <0.01 |
| ***4. basal inferior*** | 0.72  (0.48-0.85) | <0.001 | 0.52  (-0.09-0.85) | 0.05 | 0.74  (0.25-0.93) | <0.01 | 0.41  (-0.32-0.82) | 0.12 |
| ***5. basal posterior*** | 0.75  (0.54-0.87) | <0.001 | 0.63  (0.08-0.89) | 0.02 | 0.97  (0.87-0.99) | <0.001 | 0.33  (-0.25-0.77) | 0.14 |
| ***6. basal lateral*** | 0.67  (0.41-0.83) | <0.001 | 0.37  (-0.37-0.80) | 0.15 | 0.91  (0.67-0.98) | <0.001 | 0.42  (-0.15-0.81) | 0.80 |
| ***7. mid anterior*** | 0.86  (0.72-0.93) | <0.001 | 0.79  (0.38-0.94) | <0.01 | 0.40  (-0.28-0.81) | 0.12 | 0.81  (0.43-0.95) | 0.001 |
| ***8. mid anteroseptal*** | 0.67  (0.41-0.83) | <0.001 | 0.88  (0.60-0.97) | <0.001 | 0.24  (-0.44-0.74) | 0.25 | 0.36  (-0.38-0.80 | 0.15 |
| ***9. mid septal*** | 0.76  (0.54-0.88) | <0.001 | 0.44  (-0.27-0.83) | 0.10 | 0.85  (0.50-0.96) | <0.001 | 0.66  (0.14-0.90) | 0.01 |
| ***10. mid inferior*** | 0.56  (0.25-0.76) | 0.001 | 0.51  (-0.06-0.84) | 0.04 | 0.76  (0.26-0.93) | <0.01 | 0.06  (-0.43-0.60) | 0.43 |
| ***11. mid posterior*** | 0.79  (0.60-0.90) | <0.001 | 0.74  (0.22-0.93) | <0.01 | 0.71  (0.20-0.92) | <0.01 | 0.52  (-0.05-0.85) | 0.02 |
| ***12. mid lateral*** | 0.61  (0.32-0.79) | <0.001 | 0.49  (-0.21-0.85) | 0.08 | 0.82  (0.42-0.95) | <0.01 | 0.31  (-0.41-0.77) | 0.19 |
| ***13. apical anterior*** | 0.74  (0.52-0.87) | <0.001 | 0.40  (-0.32-0.81) | 0.13 | 0.93  (0.73-0.98) | <0.001 | 0.62  (-0.02-0.89) | <0.01 |
| ***14. apical septal*** | 0.71  (0.48-0.85) | <0.001 | 0.33  (-0.27-0.77) | 0.15 | 0.94  (0.77-0.98) | <0.001 | 0.21  (-0.44-0.72) | 0.27 |
| ***15. apical inferior*** | 0.80  (0.62-0.90) | <0.001 | 0.38  (-0.19-0.79) | 0.10 | 0.95  (0.82-0.99) | <0.001 | 0.49  (-0.22-0.85) | 0.08 |
| ***16. apical lateral*** | 0.81  (0.63-0.90) | <0.001 | 0.02  (-0.65-0.63) | 0.48 | 0.92  (0.71-0.98) | <0.001 | 0.66  (0.13-0.90) | 0.01 |
| ***Mean*** | 0.93  (0.78-0.97) | <0.001 | 0.78  (0.36-0.94) | <0.01 | 0.94  (0.76-0.98) | <0.001 | 0.68  (-0.08-0.93) | <0.001 |

Cardiovascular magnetic resonance imaging myocardial feature-tracking with mid-wall contours (CMR-FT_mid_), intraclass correlation coefficient (ICC), systolic circumferential strain (SCS), 95%-confidence interval (95%-CI), left bundle branch block patients (LBBB), hypertrophic cardiomyopathy patients (HCM)

Additional file 1. Table F: Intra-observer variability CMR-FT_mid_, ICC of T2P-SCS

|  | **Total study group**  **(n = 30)** | | **Healthy volunteers (n = 10)** | | **LBBB (n = 10)** | | **HCM (n = 10)** | |
| --- | --- | --- | --- | --- | --- | --- | --- | --- |
|  | ***ICC***  ***(95%-CI)*** | ***p-value*** | ***ICC***  ***(95%-CI)*** | ***p-value*** | ***ICC***  ***(95%-CI)*** | ***p-value*** | ***ICC***  ***(95%-CI)*** | ***p-value*** |
| ***1. basal anterior*** | 0.25  (-0.13-0.56) | 0.09 | 0.89  (0.64-0.97) | <0.001 | -0.06  (-0.69-0.57) | 0.57 | 0.03  (-0.68-0.64) | 0.47 |
| ***2. basal anteroseptal*** | 0.43  (0.11-0.68) | <0.01 | 0.87  (0.55-0.97) | <0.001 | 0.66  (0.12-0.90) | <0.01 | -0.02  (-0.54-0.57) | 0.53 |
| ***3. basal septal*** | 0.30  (-0.06-0.60) | 0.05 | -0.06  (-0.33-0.42) | 0.61 | 0.46  (-0.25-0.84) | 0.09 | -0.31  (-0.86-0.41) | 0.80 |
| ***4. basal inferior*** | 0.13  (-0.24-0.46) | 0.25 | -0.60  (-0.94-0.09) | 0.98 | -0.10  (-0.71-0.55) | 0.61 | 0.19  (-0.17-0.64) | 0.18 |
| ***5. basal posterior*** | 0.59  (-0.30-0.78) | <0.001 | 0.21  (-0.26-0.69) | 0.21 | 0.96  (0.85-0.99) | <0.001 | -0.14  (-0.65-0.49) | 0.67 |
| ***6. basal lateral*** | 0.43  (0.08-0.68) | <0.01 | 0.89  (0.61-0.97) | <0.001 | -0.23  (-0.63-0.39) | 0.80 | 0.33  (-0.19-0.76) | 0.12 |
| ***7. mid anterior*** | 0.23  (-0.14-0.55) | 0.11 | 0.53  (-0.13-0.86) | 0.05 | 0.34  (-0.35-0.78) | 0.16 | -0.07  (-0.76-0.59) | 0.57 |
| ***8. mid anteroseptal*** | 0.04  (-0.30-0.38) | 0.42 | 0.82  (0.37-0.95) | <0.001 | -0.10  (-0.44-0.44) | 0.66 | -0.05  (-0.73-0.59) | 0.56 |
| ***9. mid septal*** | 0.07  (-0.30-0.42) | 0.35 | 0.54  (-0.13-0.87) | 0.05 | 0.12  (-0.55-0.68) | 0.36 | -0.16  (-0.82-0.53) | 0.66 |
| ***10. mid inferior*** | 0.71  (0.47-0.48) | <0.001 | 0.33  (-0.38-0.78) | 0.17 | 0.74  (0.23-0.93) | <0.01 | 0.66  (0.07-0.91) | 0.02 |
| ***11. mid posterior*** | 0.64  (0.37-0.81) | <0.001 | 0.51  (-0.12-0.85) | 0.06 | 0.47  (-0.12-0.83) | 0.07 | 0.74  (0.27-0.93) | <0.01 |
| ***12. mid lateral*** | 0.88  (0.77-0.94) | <0.001 | 0.67  (0.13-0.91) | 0.01 | 0.88  (0.60-0.97) | <0.001 | 0.80  (0.39-0.95) | <0.01 |
| ***13. apical anterior*** | 0.51  (0.20-0.73) | 0.001 | 0.76  (0.32-0.94) | <0.01 | 0.53  (-0.03-0.85) | 0.03 | -0.16  (-0.80-0.53) | 0.66 |
| ***14. apical septal*** | 0.57  (0.26-0.77) | 0.001 | 0.02  (-0.69-0.64) | 0.48 | 0.87  (0.59-0.97) | <0.001 | 0.29  (-0.25-0.74) | 0.16 |
| ***15. apical inferior*** | 0.77  (0.57-0.88) | <0.001 | 0.81  (0.40-0.95) | 0.001 | 0.81  (0.40-0.95) | <0.01 | 0.53  (-0.03-0.85) | 0.03 |
| ***16. apical lateral*** | 0.62  (0.34-0.80) | <0.001 | 0.85  (0.49-0.96) | 0.001 | 0.47  (-0.21-0.84) | 0.08 | 0.64  (0.07-0.89) | 0.02 |
| ***Mean*** | 0.75  (0.54-0.87) | <0.001 | 0.71  (0.17-0.92) | <0.01 | 0.51  (-0.06-0.84) | 0.02 | 0.60  (-0.04-0.88) | 0.03 |

Cardiovascular magnetic resonance imaging myocardial feature-tracking with mid-wall contours (CMR-FT_mid_), intraclass correlation coefficient (ICC), time to systolic circumferential strain (T2P-SCS), 95%-confidence interval (95%-CI), left bundle branch block patients (LBBB), hypertrophic cardiomyopathy patients (HCM)

Additional file 1. Table G: Inter-observer variability of CMR-FT_mid_, ICC of T2P-SCS

|  | **Total study group**  **(n = 30)** | | **Healthy volunteers (n = 10)** | | **LBBB (n = 10)** | | **HCM (n = 10)** | |
| --- | --- | --- | --- | --- | --- | --- | --- | --- |
|  | ***ICC***  ***(95%-CI)*** | ***p-value*** | ***ICC***  ***(95%-CI)*** | ***p-value*** | ***ICC***  ***(95%-CI)*** | ***p-value*** | ***ICC***  ***(95%-CI)*** | ***p-value*** |
| ***1. basal anterior*** | 0.58  (0.28-0.77) | <0.001 | 0.89  (0.61-0.97) | <0.001 | 0.16  (-0.59-0.71) | 0.33 | 0.80  (0.36-0.95) | 0.001 |
| ***2. basal anteroseptal*** | 0.15  (-0.20-0.47) | 0.21 | 0.92  (0.74-0.98) | <0.001 | 0.55  (-0.01-0.86) | 0.03 | -0.31  (-0.83-0.39) | 0.81 |
| ***3. basal septal*** | -0.06  (-0.41-0.31) | 0.62 | -0.10  (-0.47-0.45) | 0.66 | -0.39  (-0.94-0.36) | 0.84 | 0.08  (-0.61-0.66) | 0.42 |
| ***4. basal inferior*** | 0.33  (-0.05-0.61) | 0.05 | 0.24  (-0.34-0.72) | 0.22 | -0.02  (-0.61-0.59) | 0.52 | 0.23  (-0.52-0.74) | 0.27 |
| ***5. basal posterior*** | 0.47  (0.13-0.71) | <0.01 | 0.30  (-0.36-0.77) | 0.19 | 0.45  (-0.27-0.83) | 0.09 | 0.43  (-0.12-0.81) | 0.06 |
| ***6. basal lateral*** | 0.65  (0.38-0.82) | <0.001 | 0.83  (0.46-1.00) | 0.001 | 0.77  (0.35-0.94) | <0.01 | 0.35  (-0.37-0.79) | 0.16 |
| ***7. mid anterior*** | 0.47  (0.14-0.71) | <0.01 | 0.77  (0.31-0.94) | <0.01 | 0.41  (-0.29-0.81) | 0.12 | 0.49  (-0.19-0.85) | 0.07 |
| ***8. mid anteroseptal*** | 0.34  (<0.01-0.62) | 0.03 | -0.11  (-0.75-0.56) | 0.61 | 0.19  (-0.23-0.66) | 0.23 | 0.76  (0.32-0.93) | <0.01 |
| ***9. mid septal*** | 0.10  (-0.28-0.44) | 0.30 | 0.51  (-0.19-0.86) | 0.07 | 0.62  (0.08-0.89) | 0.02 | -0.68  (-1.07-0.04) | 0.97 |
| ***10. mid inferior*** | 0.72  (0.49-0.86) | <0.001 | 0.82  (0.46-0.95) | 0.001 | 0.87  (0.55-0.97) | <0.001 | 0.25  (-0.44-0.74) | 0.24 |
| ***11. mid posterior*** | 0.75  (0.54-0.87) | <0.001 | 0.84  (0.47-0.96) | 0.001 | 0.61  (0.06-0.88) | 0.02 | 0.71  (0.23-0.92) | <0.01 |
| ***12. mid lateral*** | 0.78  (0.60-0.89) | <0.001 | 0.38  (-0.30-0.80) | 0.13 | 0.80  (0.42-0.95) | 0.001 | 0.59  (-0.04-0.88) | 0.03 |
| ***13. apical anterior*** | 0.44  (0.11-0.69) | <0.01 | 0.66  (0.08-0.91) | 0.02 | 0.29  (-0.37-0.76) | 0.20 | <-0.01  (-0.70-0.62) | 0.50 |
| ***14. apical septal*** | 0.44  (0.11-0.68) | <0.01 | 0.55  (-0.13-0.87) | 0.05 | 0.47  (-0.15-0.84) | 0.07 | 0.32  (-0.22-0.76) | 0.14 |
| ***15. apical inferior*** | 0.88  (0.77-0.94) | <0.001 | 0.80  (0.37-0.95) | 0.001 | 0.88  (0.56-0.97) | <0.001 | 0.83  (0.47-0.95) | 0.001 |
| ***16. apical lateral*** | 0.38  (0.05-0.64) | 0.01 | 0.89  (0.62-0.97) | <0.001 | 0.27  (-0.32-0.74) | 0.20 | 0.24  (-0.45-0.74) | 0.25 |
| ***Mean*** | 0.77  (0.57-0.88) | <0.001 | 0.83  (0.47-0.95) | 0.001 | 0.48  (-0.08-0.83) | 0.03 | 0.73  (0.20-0.93) | <0.01 |

Cardiovascular magnetic resonance imaging myocardial feature-tracking with mid-wall contours (CMR-FT_mid_), intraclass correlation coefficient (ICC), time to systolic circumferential strain (T2P-SCS), 95%-confidence interval (95%-CI), left bundle branch block patients (LBBB), hypertrophic cardiomyopathy patients (HCM)
